# Exploration of the Conditions for Occurrence of Photoplethysmographic Signal Inversion above the Dorsalis Pedis Artery

**DOI:** 10.3390/s24206505

**Published:** 2024-10-10

**Authors:** Fredrik Wilsbeck Jerve, Dag Roar Hjelme, Håvard Kalvøy, John Allen, Christian Tronstad

**Affiliations:** 1 Department of Electronic Systems, Norwegian University of Science and Technology, 7034 Trondheim, Norway; dag.hjelme@ntnu.no; 2 Department of Clinical and Biomedical Engineering, Oslo University Hospital, 0372 Oslo, Norway; hkalvoy@ous-hf.no; 3 Research Centre for Intelligent Healthcare, Coventry University, Coventry CV1 5RW, UK; ad5325@coventry.ac.uk

**Keywords:** artery, cardiovascular disease, PPG, photoplethysmography, peripheral vascular disease, pulse, pulse wave analysis, PPG inversion

## Abstract

Inversion of the photoplethysmographic (PPG) signal is a rarely reported case. This signal anomaly can have implications for PPG-based cardiovascular assessments. The conditions for PPG signal inversion in the vicinity of the dorsalis pedis (DPA) artery of the foot were investigated. Wireless multi-wavelength PPG sensing with skin-probe contact pressure and local skin temperature were studied at different sensor positions, and the occurrence of inversion (OOI) was investigated. Twelve healthy adult volunteers were studied over four LED wavelengths at three levels of contact pressure for 11 probe positions. A novel algorithm quantified the proportion of inverted samples with respect to the abovementioned variables. Our algorithm classifying inverted vs. non-inverted pulses achieved 98.3% accuracy. Ten of the participants had at least one inverted signal identified. The impact of interindividual variation on inversion prevalence was large, but different LEDs, relative position to the DPA and sensor contact pressure also affected OOI. Skin surface and room temperatures showed no impact on OOI. Lateral measurements showed 39.6% more inversion at maximum compared to minimum contact pressure. Mechanical capillary bed variations and arterial reflections during venous engorgement are considered viable explanations for our observations. These findings motivate an expanded study of the occurrence of PPG signal inversion.

## 1. Introduction

Photoplethysmography (PPG) is an optical technique for detecting the changes in perfusion in the peripheral vascular bed of tissue [1]. The first paper discussing monitoring of peripheral circulation with light was as early as the mid-1930s by Molitor and Kniazuk [2]. It was later applied by Hertzman [3] in finger and septum measurements, from which the term “photoelectric plethysmography” first emerged. Since then, PPG has set its mark both in clinical practice and consumer electronics products due to its simplicity, low cost, non-invasiveness, and capability for miniaturization. Thus, PPG is already integrated into numerous smartwatches mainly for heart rate tracking, but is also applicable for peripheral vascular assessment, such as diagnosis of arterial incompetence and venous refilling time [4].

The PPG signal can be characterized by the detector (e.g., photodiode) light intensity level around which it oscillates (“DC”) and the size and phase of the oscillations (“AC”). The DC level (quasi-static) stems from the low-frequency optical absorbers and optical scatterers in tissue, such as muscle, tendons, bone, non-pulsatile venous/arterial blood, and more. The higher frequency variations are thought to represent a number of mechanisms, including the orientation and deformation of the red blood cells, distribution and variation of absorbers and blood volumes, and lastly movement of the capillary bed [5]. For shorter blue wavelengths the modulation of the non-vascularized epidermis is suspected to be of importance [6].

For the vast majority of papers using reflectance PPG, the intensity of the reflected light decreases with the systolic phase. However, PPG signal inversion is a rarely reported pulse signal anomaly where systolic volumes increase the observed optical intensity. The following phase, commonly associated with diastolic relaxation leads to an intensity decline. The result is thus a flipped/inverted signal shape in the same light intensity direction as the arterial blood pressure curve and the opposite of what is to be expected for reflectance PPG. The shape of the pulse signal may then complicate the signal processing for extraction of various features of the PPG signal, and potentially impact the PPG measurement reproducibility.

For inversion to be possible, there must be a change in the optical properties of the skin measurement site. Previous studies have discussed inverted signals in relation to various variables related to PPG measurements. Among these are contact pressure [7], position of the PPG probe [8,9,10], and wavelength [9]. A single protocol study thus seems valuable. Since the inverted pulses still follow the heartbeat frequency, we believe the mechanism behind inversion must be related to local changes in the vascular bed and/or surrounding tissue.

Weinman et al. [11] hypothesized that inversion was to occur when reflection from vessel expansions would dominate the attenuated backscattered and reflected light from the surrounding tissue. Nijboer and Dorlas [7] suggested that reflections from arteries under heightened optical densities of the surrounding tissue from venous engorgement, in combination with applied pressures, caused inversion. The Kamshilin et al. [9] model of green light reflection PPG involves probing of transmural pressure-induced density changes in the capillary bed. Inversion could therefore occur when measuring adjacent, counter-phase areas to these density changes. Amelard et al. [12] observed inversion during contactless PPG imaging of the jugular venous pulse, and the inversion was credited to the veins’ differential pressure profiles relative to the carotid. Long and Wan-Young Chun [10] discovered two types of PPG signals with opposite polarities during wrist measurements. They thought tissue mechanical changes by heart operations and external pressure, relatively increasing light reflection off erythrocytes, caused inversion. Choi et al. [13] observed inversion during measurements of the radial artery whenever participants rotated their wrists. Signal shape distortion was ascribed to positional offset and applied pressure of the PPG sensor during wrist motion. Bonnet et al. [8] observed inversion whenever the sensor was placed at the site where the carotid artery pulsation was strongest. They also observed that the systolic upstroke was steeper in inverted pulses relative to non-inverted pulses.

In summary, signal inversion is known to occur, especially for reflective mode PPG, and seems to be dependent on positioning relative to an artery, wavelength, and sensor contact pressure. Applications of PPG, such as averaged heart rate measurement, are quite robust against signal distortion. However, applications using pulse signal characteristics that require more precise determination of fiducial points (e.g., pulse onset, dicrotic notch, and maximum derivatives) may be susceptible to errors from inversion, depending on the site of measurement. Considering the popular recent application of PPG for non-invasive blood pressure prediction based on the PPG signal characteristics and/or the pulse wave velocity estimated by the time difference between fiducial points from two PPG sensors [14], its performance relies on undistorted signals. Due to its relevance for peripheral arterial stiffness measurement, we have studied the conditions for PPG signal inversion measuring above the dorsalis pedis artery. This artery is the prime source of perfusion to the dorsum of the foot and has a diameter of about 2–3 mm depending on gender and the site of measurement. This artery is also relevant for peripheral artery disease diagnosis, including people with diabetes, where palpation of the pedal artery is a common test [15], but with room for improvement in interobserver reliability [16]. Objective sensor measurements may improve reliability, but this relies on reproducible and unconfounded signals reflecting the properties of this artery, for which we believe that understanding the conditions for signal distortion is important.

Thus, the aim of this study was to investigate the conditions for the occurrence of PPG signal inversion when measuring at the skin surface close to a main artery on the foot (Dorsalis Pedis Artery, DPA). PPG signal inversion may be a rare phenomenon with respect to current clinical applications, as we are, for instance, not aware of reports on PPG inversion on the fingertips. However, there are certain potential clinical applications of PPG where we believe this phenomenon to be more likely to occur and to be worth investigating, such as local pulse wave velocity measurement along specific arteries, which is also relevant toward cuffless blood pressure estimation. Investigations of the conditions for PPG inversion will let us identify the conditions for the occurrence of this signal distortion, quantify potential measurement error and implications, and potentially improve the technology of future clinical or consumer applications of PPG.

To our knowledge, this is the first study to explore the conditions for reflective PPG signal inversion when measuring above this artery for pulse wave analysis in the feet. More specifically, the goal was to quantify the influence of selected factors (sensor positioning relative to the artery, wavelength, and contact pressure) in order to further develop the understanding of the phenomenon to support the improvement of future applications of PPG.

## 2. Materials and Methods

### 2.1. PPG Measurement System

**Figure 1:**
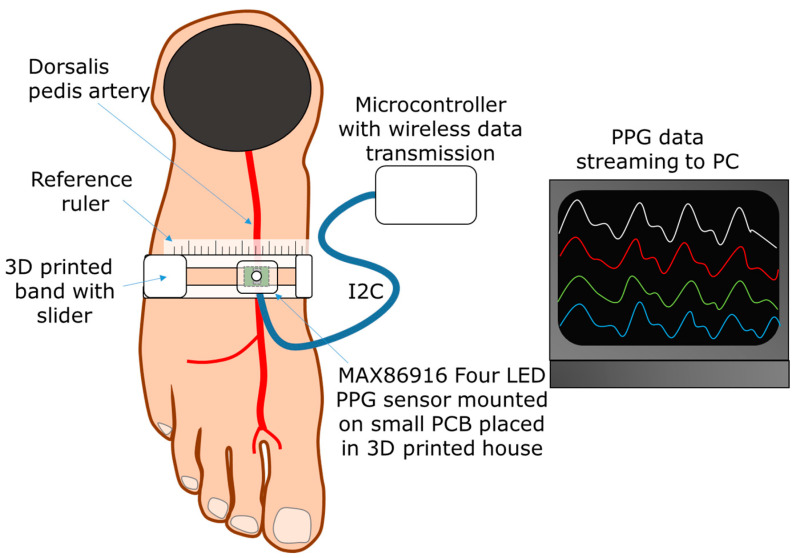
Illustration of the PPG-based measurement system used in the study. Colors on the PC (right) represents the 4 wavelengths (ir, red, green and blue) emitted during acquisition.

Figure 1 provides an overview of how the PPG signals were recorded during experiments with equipment developed for this particular study. For PPG measurements, we used a MAX86916 multi-wavelength (IR: 930–955 nm, red: 655–663 nm, green: 520–535 nm, blue: 455–466 nm) 24.5 mm^2^ integrated optical sensor platform (Analog Devices Inc., Wilmington, MA, USA [17]) controlled by an ESP32 microcontroller (Adafruit HUZZAH32, Adafruit Industries, New York, NY, USA) programmed to acquire PPG signals at 800 Hz sampling rate. The data were continuously streamed via Bluetooth to a laptop computer running a custom-made monitoring and data capture program made in LabVIEW (National Instruments Corporation, Austin, TX, USA). The PPG sensor provided multi-wavelength measurements in a sequence of the four LEDs, where each LED had a pulse width of 70 µS and a pulse amplitude of approximately 50 mA. The estimated pulse radiant powers of the four LEDs were 30, 16, 15, and 25 mW for the IR, R, G, and B LEDs respectively, resulting in a total average radiant power of 4.7 mW (four 70 µs pulses at 800 Hz rate). Assuming an average illumination area of 10 mm^2^ and a skin reflectivity of 0.5, this corresponds to a heating rate on the order of 10^−2^ °C/s (assuming tissue heat capacity of 0.7 J/°C·cm^2^), which is far below the cooling rate due to tissue blood perfusion. Therefore, the radiant power from the LEDs is expected to have a negligible effect on the measurements. The sensor was oriented in the direction of the DPA artery with LEDs positioned above the detector. As a house for the sensor and to allow fixation and sliding of the sensor with even contact pressure laterally on the dorsum of the foot, we designed and 3D printed a custom band as illustrated in Figure 1 using a combination of thermoplastic polyurethane filaments (NinjaFlex, Ninjatek Fenner Precision Polymers US and Flexfill TPU92A, Filamentum, Czech Republic). The force applied to the skin from the probe was measured locally using a thin and flexible Flexiforce A201 piezoresistive force sensor (Tekscan Inc., Norwood, MA, USA. Photo provided in Appendix A Figure A2), where the sensing area (68.7 mm^2^) is concentrated on the tip of the sensor. This allows for sensor bending without disrupting force readings. The force sensor was connected in series with a load resistor on a breadboard and a microcontroller (ESP32) with serial PC connection measured voltages over the load resistor. The force sensor was calibrated using weights of known values. In addition to force and PPG measurements, the ambient room temperature was measured with a regular thermometer having ±0.5 °C accuracy, and the mean skin surface temperatures near the DPA were measured with HikMicro HM-TP42 3AQF/W-POCKET2 (Hangzhou, China) pocket thermal imaging camera. Thermal recordings were performed to track whether measurement site temperature could affect the occurrence of inversion.

### 2.2. Protocol and Research Participants

**Figure A1:**
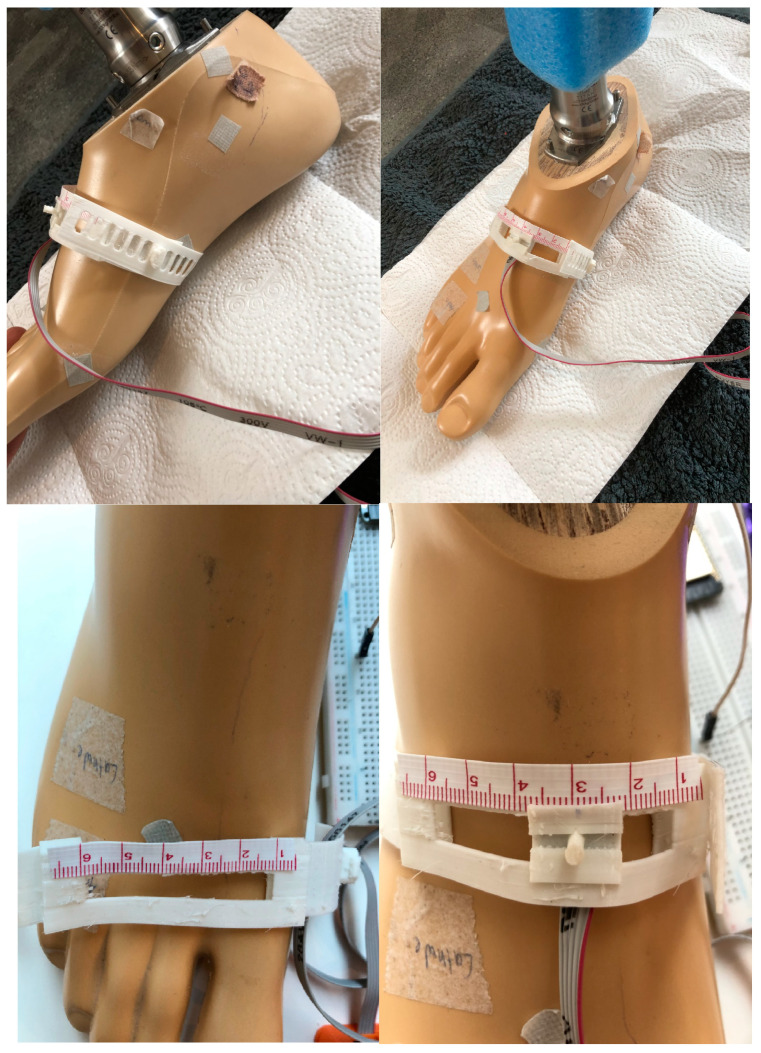
Images of measurement setup and 3D printed strap. The PPG sensor is located within the rectangular house and can be moved laterally along the mm scale.

**Figure A2:**
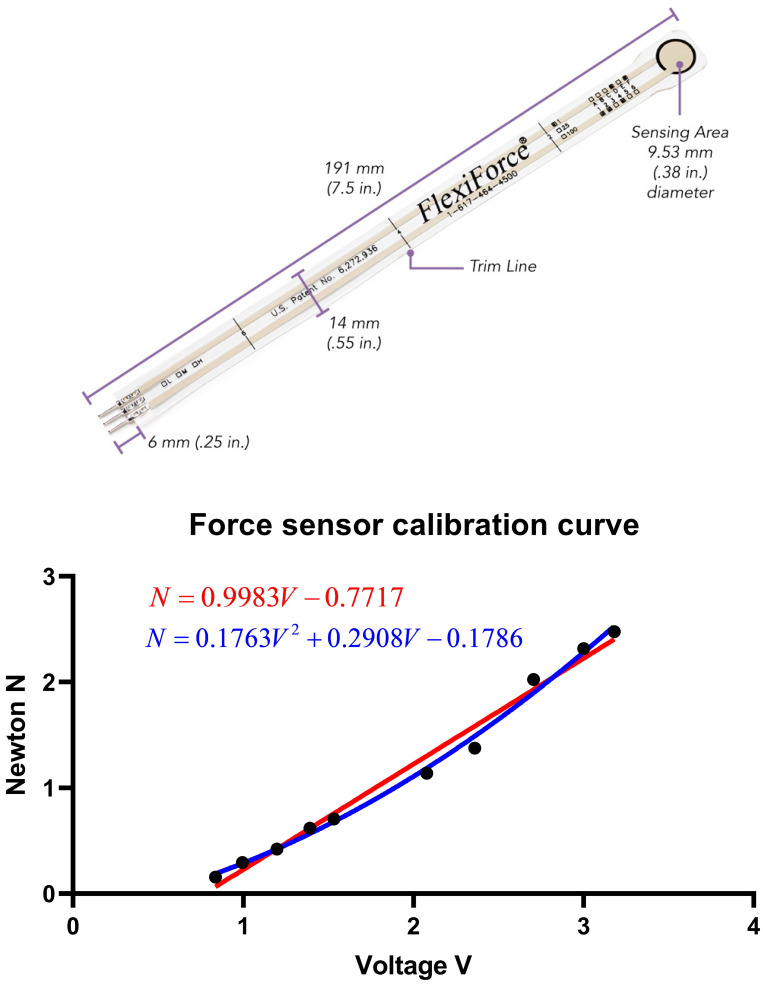
Flexiforce Tekscan A201 force sensor and calibration curve (red).

A pilot study investigating the inverted PPG signals on the dorsalis pedis artery was performed on twelve healthy adult volunteers. The biological sex distribution was 8 males and 4 females. A total of 10 out of the 12 volunteers were aged 25–28, while the final two were aged 61 and 62. All volunteers had a type 2 skin color with respect to the Fitzpatrick scale. The study was evaluated by the regional ethics committee (REK Midt, application nr 587491), and the committee concluded that the study falls outside the scope of Section 2 and Section 4 of the Health Research Act. As a result, it can proceed and be published without approval from REK. All participants gave informed consent for the study. Measurements comprised thirty-three 90 s multi-wavelength reflection recordings spanning 1 cm across the perceived location of the DPA pulse. Study participants were not allowed to consume caffeine or alcohol in the last 24 h before the measurements. The study coordinator (FWJ) prepared the measurement facility and environment with respect to variables that could affect PPG measurements: room temperature, ambient lighting, and circulating draught. During measurement, the participants sat in a comfortable chair with their right foot grounded on the floor on top of a towel with an approximate 90° knee angle. Once informed consent was obtained and the study participant had acclimatized to the room environment, measurements were initiated by locating the DPA by palpation followed by IR camera images to record the skin surface temperature distribution. Then, the 3D-printed band was fastened around the right foot with the PPG sensor centered on top of the DPA location. The strap was initially set to the maximum level of pressure that was still comfortable for the volunteer using the different levels of tightness that the 3D-printed band allowed. The levels of contact pressure against the skin were measured before starting each round of PPG measurements. Each PPG recording was over 90 s, with constant visual monitoring of sensor output throughout. The first recording was with the sensor placed on the origin defined as directly above of the perceived peripheral pulse. The sensor position was then moved in 1 mm increments medially along the strap for five consecutive measurements until the 5 mm medial position, followed by another five consecutive medial measurements where the probe was moved 6 mm laterally to measure 1 to 5 mm laterally to the DPA (see Figure A1 in Appendix A for details). Table 1 shows the schedule of measurements for each participant totaling 11 different positions repeated over 3 levels of pressure. The force measured from the sensor on the skin at the different pressure levels for all the participants, along with the skin temperature measured at the dorsal skin of the foot is summarized in Table 2. A single operator (FWJ) performed all of the PPG/study measurements.

### 2.3. PPG Signal Processing

**Figure 2:**
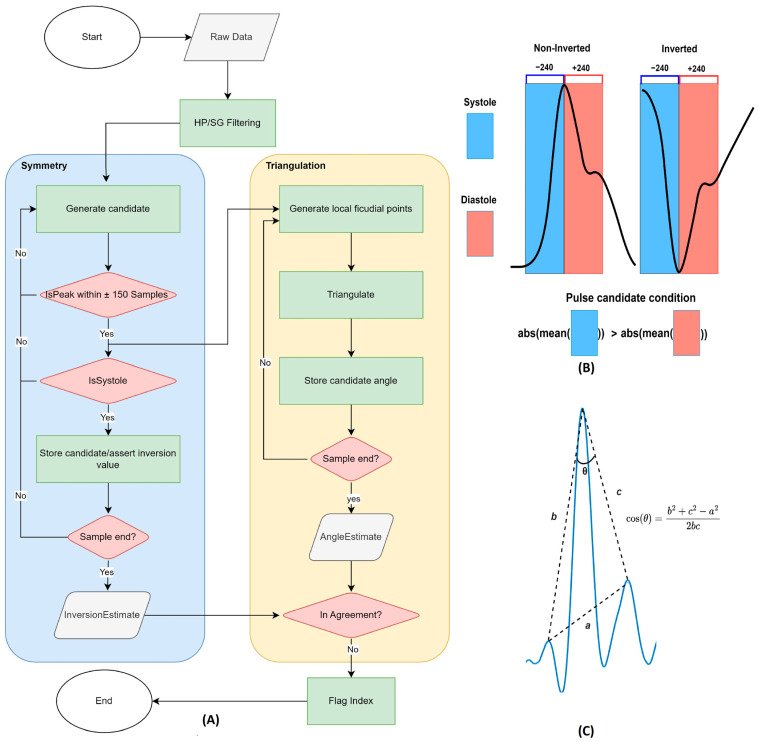
(**A**) Inversion algorithm flowchart and logic representation. (**B**) Systole/diastole asym-metry check. Blue and red shaded areas indicate the systolic and part of the diastolic phase in a PPG signal. In this case, both the non-inverted and the inverted signals fulfill the systole/diastole symmetry condition since their deflections are steepest in the previous 240 samples. (**C**) Triangulation with adjacent local peaks for pulse candidate sharpness evaluation. Sharp candidates are thought to represent systolic deflections. The angle theta of the constructed triangle is calculated by the law of cosine [13].

Signal processing was performed using MATLAB (R2023 A Mathworks Inc.). Each recording was first negated to match the more familiar arterial blood pressure (ABP) waveform, and an inverted PPG signal would then be inversely related to the ABP curve. In order to quantify the degree of inversion (percent of inverted pulses in a time series), inversion estimates were generated through a novel algorithm evaluating symmetry and sharpness of signal extreme points which may represent the systolic end of a single pulse candidate. Noise components were first suppressed through high-pass and low-pass filtering. The high pass filter had a stopband attenuation of 60 dB and a passband frequency of 0.1 Hz. The Savitzsky-Golay low-pass filter had a filter order of 20 and a frame length of 999. An algorithm was then employed to evaluate the pulse candidates (Figure 2A), where the first layer locates peaks and troughs to gather all possible PPG pulses, as illustrated in Figure 2B, using the end of the systole as reference points for every pulse. Peaks were located through the findpeaks() function in Matlab, and each reference point was verified by comparing its value with the nearest 150 samples. If any neighboring points were either greater or lower than the estimated peak/trough, they were removed from the systolic end/pulse candidate list. For a peak (non-inverted) or trough (inverted) to represent the end of systole, the PPG photodiode sensor should observe a rapid change in intensity representing the systolic contraction, followed by a gradual diastolic increase or decrease. Pulse candidates not fulfilling this symmetry are unlikely to represent a clean PPG pulse and were thus removed from the candidate list. Systole/diastole symmetry was translated to Matlab pulse wave analysis code by comparing the tangent (the absolute mean first order derivatives) during the end systole and beginning of diastole using a window of 240 samples (approximately 0.3 s) before and after the reference point. See Figure 2A for a flowchart and Figure 2B for a visual representation of the symmetry algorithm.

**Figure A3:**
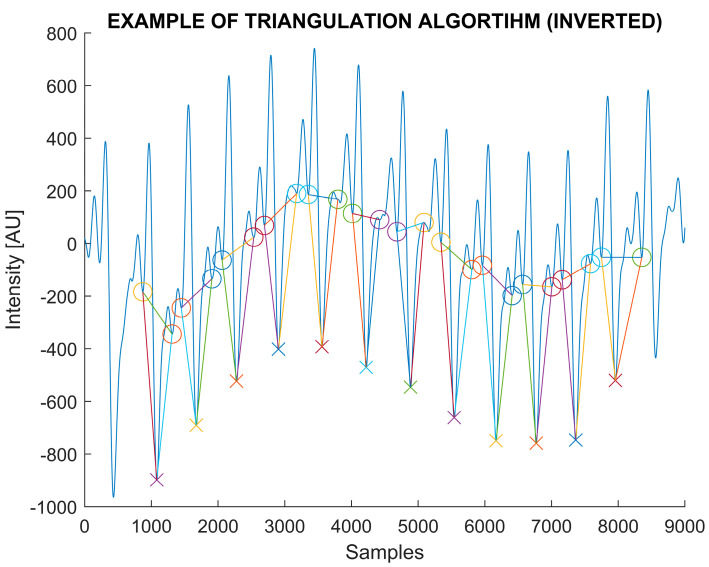
Example of Triangulation algorithm for angle estimation for an inverted signal. Angle estimates are acute and agree with the located inverted pulse candidates from the symmetry algorithm. The inversion estimate was −0.9487 for this signal. The *Y*-axis is post SG/HP filtering and is negated. Crosses are systolic end points, while circles are adjacent troughs located by the algorithm.

**Figure A4:**
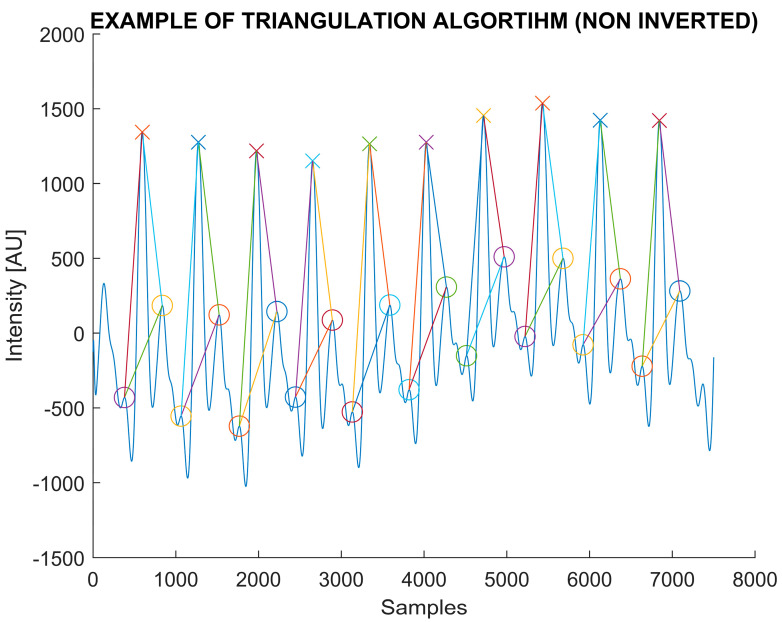
Example of Triangulation algorithm for angle estimation for a non-inverted signal. Angle estimates are acute and agree with the located non-inverted pulse candidates from the symmetry algorithm, Inversion estimate was 0.96 for this signal. The *Y*-axis is post SG/HP filtering and is negated. Crosses are systolic end points, while circles are adjacent peaks located by the algorithm.

**Figure A5:**
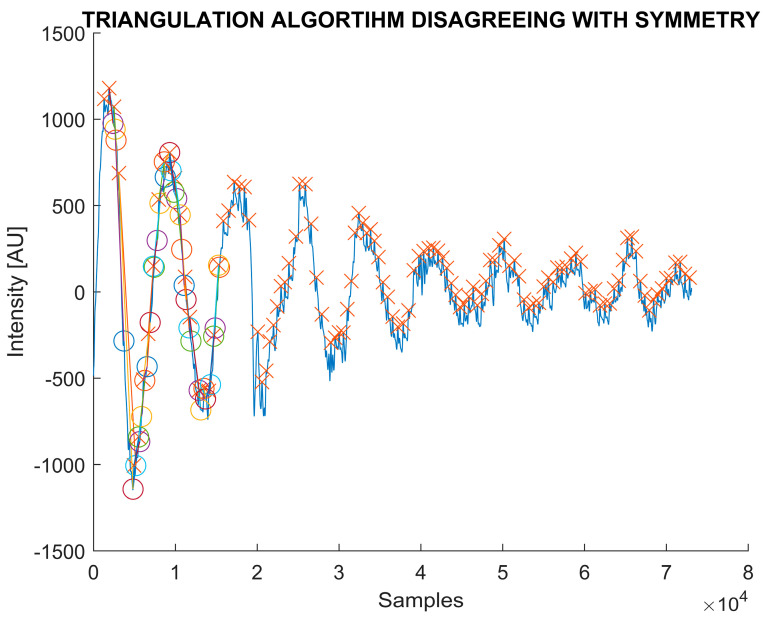
A 90 s recording example where the triangulation algorithm disagrees with the symmetry algorithm. X-marks refers to pulse candidates located by the symmetry algorithm (small peaks). Triangulation was stopped for easier interpretation. The symmetry algorithm will consider this a non-inverted signal; however, this sample is distorted, causing the initial angle estimates to be obtuse and the sample is thus flagged for investigation. The inversion estimate is 0.80 for this signal. The *Y*-axis is post SG/HP filtering and is negated. Crosses are systolic end points, while circles are adjacent peaks located by the algorithm.

Peaks and troughs satisfying the symmetry condition were labeled in a separate matrix with either the value 1 for peaks (non-inverted) or −1 for troughs (inverted) pulses. The mean of this matrix is used to indicate the degree of inversion of the sample. A mean close to 1 indicates a consistently non-inverted signal, whereas −1 implies a consistently inverted signal. Values near zero either suggest sections of both inverted and non-inverted pulses during the time series or a noise-corrupted signal. The second layer of the algorithm (Triangulation) evaluates the sharpness of the systolic upstroke, as illustrated in Figure 2C. In theory, the systole should produce greater deflections than other sources of a PPG signal. This translates to sharper, or more acute angles during triangulation [13]. Pulse candidates were triangulated with neighboring local (non-pulse candidate) maximums and minimum points. If the pulse candidate is a trough, it is triangulated with neighboring minimum points, and peak pulse candidates are triangulated with adjacent maximum points. As shown in Figure 2C, the length of vectors (a, b, and c) between these points was calculated based on the temporal (x) and PPG intensity (y) axes. Angles (θ) were calculated by the law of cosine, where acute angles produce positive values, and obtuse angles produce negative values of cosine of θ. An inverted sample should have more acute angles generated for its troughs rather than its peaks, and this implies that the mean angle of peaks and troughs throughout a recording provides insight into the signal phase. While the first layer evaluates symmetry, this mean angle evaluates sharpness and was used as the second criterion to quantify PPG pulse inversion. If the two criteria were to disagree on the sample phase, it was flagged for investigation, and each recording was manually inspected visually by one of the Authors (FWJ). Examples of algorithm operation are shown in Appendix A in Figure A3, Figure A4 and Figure A5. Noise-corrupted signals with either unrecognizable pulses or where AC variations were challenging to separate from other deflections, such as motion artifacts, were excluded from the analysis. Red wavelengths at low pressures were most affected amounting to 49.3% of the discarded data. In total, 284 out of the 1584 recordings (12 participants × 11 positions × 3 pressures × 4 wavelengths) were considered unfit for analysis. Since corrupted signals are unlikely to be consistent in inversion estimation, their degree of inversion is expected to be close to 0. In a separate noise detection test, the algorithm had an 89.96% success rate in detecting faulty signals when comparing signals with less than 0.3 degrees of inversion (DOI) and known corrupted signals from visual inspection. The algorithm had a 98.3% success rate in correctly labeling a signal inverted/noninverted with 0.54% false negatives (labeled non-inverted, when actually inverted), and 1.07% false positives (labeled inverted when actually non-inverted) when excluding corrupted signals. A total of 94.8% of the inverted samples were correctly detected as inverted.

### 2.4. Statistical Analysis 

The main statistical parameter utilized was the mean occurrence of inverted pulses. This mean is calculated over every 90 s PPG recording by summing each binary pulse contribution according to inversion degree, and then averaging over the total number of pulses in the recording as given in Equation (1).

(1) $$\mu_{inversion}=\frac{1}{N_{pulses}}\sum_{i=1}^{i=N_{pulses}}a_{i},\;where\;a_{i}{=}_{-1\;if\;inverted}^{1\;if\;non\;inverted}$$

With this mean value *µ_inversion_*, the degree of inversion (DOI) of a sample was then defined as in Equation (2).

(2) $$DOI\;=\;\frac{1-\mu_{inversion}}{2}$$

**Figure A6:**
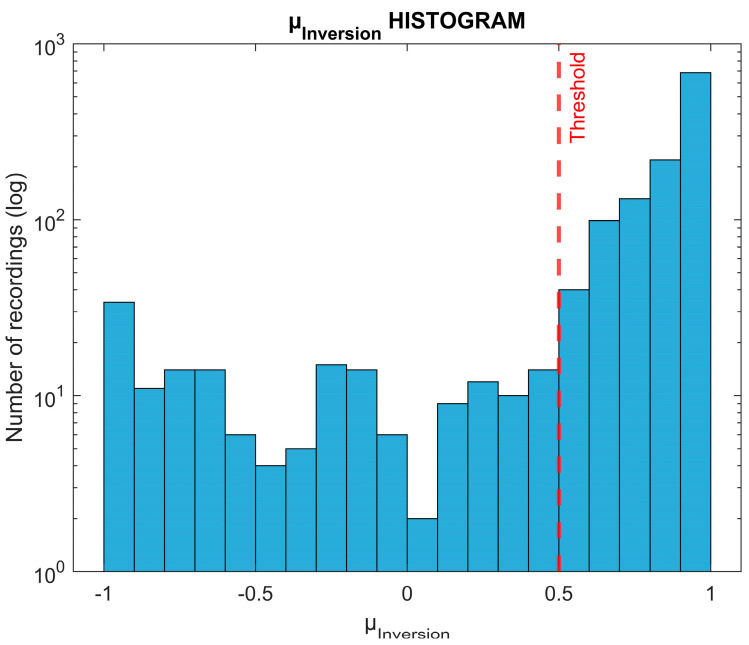
Histogram of all recordings mean degree of inversion *µ_Inversion_* in logarithmic scale. Noisy or corrupted PPG signals are excluded. With a threshold of *µ_Inversion_* < 0.5 (equal to DOI > 0.25), around 12.65% of all recordings were considered inverted.

A sample with DOI of 0 is then completely non-inverted, while a DOI of 1 is inverted. Once all recordings have their DOI estimate, measurements with identical configurations (pressure, wavelength, and position) were grouped to study the impact of these factors on the occurrence of inversion (OOI). Since the aim of this study was to assess the conditions of inversion, all samples with a DOI > 0.25 (25% of pulses in 1 sample are inverted, equal to *µ_Inversion_* < 0.5) were considered to be significantly inverted in a binary classification. See Figure A6 in Appendix A for information on threshold selection. Calculation of OOI was performed by dividing the number of recordings with DOI > 0.25 (indicating a significant degree of inversion) by the total number of measurements at the same configuration as in Equation (3).

(3) $$OOI\;=\;\frac{1}{N_{recordings}}\sum_{j=1}^{j=N_{recordings}}b_{j},\;where\;b_{j}{=}_{0\;otherwise}^{1\;if\;DOI\;>\;0.25}$$

These parameters provide a quantified indication to which extent an individual sample is inverted (*µ_inversion_*) in a 0 to 1 scale (DOI) such that the impact of specific measurement configurations can be compressed into one (OOI), given a specific inversion threshold. For this study, OOI is the driving parameter from which results are produced.

Graphing of data was completed with GraphPad Prism (version 8.4.3), which also provided the confidence intervals of means presented in the results.

## 3. Results

**Figure 3:**
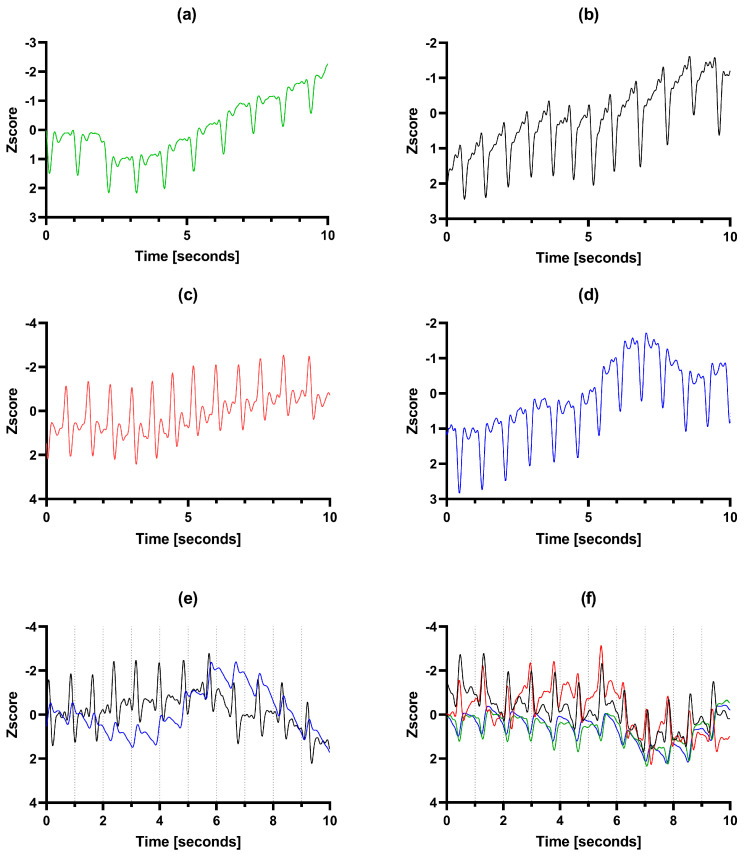
Examples (**a**–**d**) of 10 s segments of PPG recordings from different participants demonstrating signal inversion at different wavelengths. The line colors correspond to the different LEDS, where IR = black, and the vertical axes represent the negative Z score standardization of the sensor analog to digital converted value. (**e**,**f**) displays the contrast in signal shape between PPG from different LEDs measured simultaneously, caused by inversion.

PPG signal inversion occurred at least over one LED for ten of the twelve participants studied for the DPA site. Examples of inverted PPG signals for all LED wavelengths are provided in Figure 3. As shown in the Figure, inversion could occur for all LEDs/wavelengths, and it was also observed that the signal could be partly inverted with strong deflections in both directions as presented in Figure 3c,e. As shown in Figure 3f, inversion could occur for certain LEDs, while other LEDs were normal during the same measurement.

**Figure 4:**
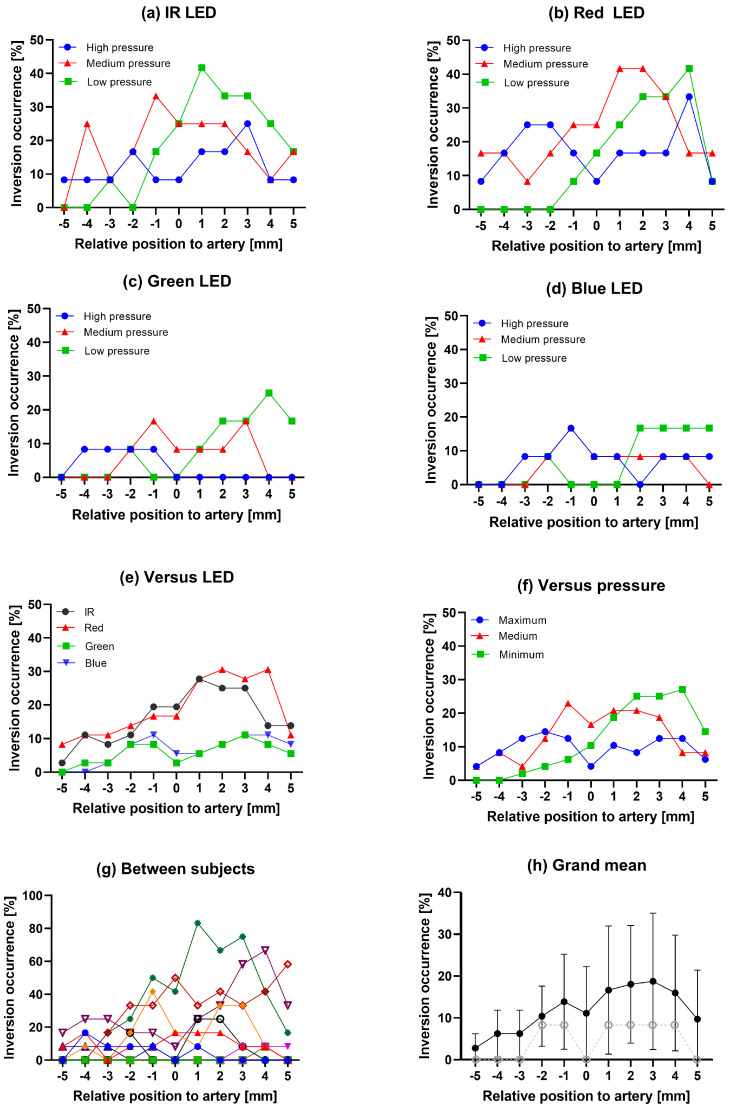
Percentwise occurrence of PPG signal inversion versus sensor position relative to the DPA for the four LEDs dependent on contact pressure (**a**–**d**), averaged over pressure levels to visualize LED dependency (**e**), averaged over LEDs to visualize pressure dependency (**f**). Averaged occurrence over all pressures and LEDs for each subject is shown in (**g**), and the grand mean (averaged over LEDs, pressures, and all participants) with the confidence interval of the mean is shown in (**h**), with the addition of the median (grey) for comparison. The positive position on the x-axis corresponds to sensor positioning in the medial direction. Color between subject (**g**).

The occurrence of inversion is represented as the portion of samples that includes inverted components relative to all samples at the specific position, wavelength, and contact force. All subfigures in Figure 4 represent the degree of inversion occurrence at each position for the specific measurement configuration explained as the following: For example, Figure 4a shows the OOI for IR LED over three pressure levels. At an arbitrary position and pressure, there are a total of twelve inversion estimates (from 12 participants). At position −5 mm and for IR LED, only one participant produced an inverted signal at the maximum probe-tissue pressure, resulting in an 8.33% (1/12) percentage occurrence of inversion.

Figure 4 summarizes these percentwise occurrences of inversion according to the different factors studied, showing variations according to sensor position, pressure, and the different LEDs. In addition, the large intersubject variability is apparent from Figure 4.

## 4. Discussion

### 4.1. Key Observations

Based on the absolute inversion estimates, the LED wavelength and source-detector distances, the applied force, and the sensor position all influence the occurrence of inverted PPG signals. Sensor location had a 15.9% inversion occurrence difference between the +3 mm and −5 mm positions. The different LEDs showed a 12.4% higher occurrence on average for the red LED compared to the green. The least influential factor was the strap tightness, with as little as a 3.6% sum difference in the occurrence of inversion between medium and maximum pressures. The contact pressure seemed to affect at which location the inversion would occur relative to the DPA. The most important observations from the main results (Figure 4) are:

- Signs of an asymmetry in inversion for lateral vs. medial measurements relative to the expected DPA position—especially for the low and medium pressure levels
- Large interindividual differences in inversion occurrence
- More occurrence of inversion for the IR and red LEDs compared to green and blue LEDs (different wavelengths and LED to detector distances)
- Large dependency on sensor location relative to artery center.

### 4.2. Inversion vs. Sensor Position

The position-dependent asymmetry observed in Figure 4 is potentially linked to the tissue morphology and blood vessel anatomy below the measurement site. Low venous pressures or volume may potentially reduce the occurrence of inversion from the lateral side of the DPA for red and IR wavelengths. From an anatomical standpoint, the medially weighted asymmetry of inversion can be explained by the involvement of the dorsalis vein if we assume that this vein was positioned medially with respect to the DPA for most participants in this study. If the formation of a strong absorbing background caused by venous engorgement is the source of inversion [7], there should be more inverted samples in this direction, independent of contact pressure. On the other hand, in the lateral direction, there may be only a network of smaller veins to create similar conditions. Here, sufficient contact pressures must be applied to form a collaborative increase in optical density in the surrounding tissue for inversion to occur. This observation is in agreement with the return of inverted samples in this direction at high applied pressures. The symmetry in inversion for maximum contact pressures may also be credited to the collapse of the underlying vein. The occurrence of inversion is thus reduced in the medial direction relative to lower probe-skin contact pressure settings.

It is important to note that the asymmetry is not statistically significant based on the 95% confidence interval seen in Figure 4h. Whether individual morphology, such as different positioning of the dorsalis vein leading to opposite positional bias, or other symmetrical effects such as mechanical deformation, causes the inverted signals is uncertain.

### 4.3. Inversion vs. Applied Probe Pressure

In Nijboer’s study [7] they reduced arm cuff pressures from systolic levels to 60 mmHg for inversion to occur. The local finger cuff pressures could range from 20 to 70 mmHg and still produce inversion with varying amplitudes. In comparison to contact pressures measured, in this study, the maximum force from the sensor surface on the skin was 55 mmHg, lower than the pressure limit during their study. Whether these pressures are comparable is uncertain due to differing measurement sites and their position relative to heart level. Measurements were performed on feet, involving relatively heightened venous volumes and hydrostatic pressures due to gravity. The veins were therefore likely more distended, and thus more susceptible to any applied contact pressure. Moreover, no systemic circulatory load comparable to the upper arm cuff was placed on participants in this study. Our measurements were also targeted directly on top of a main artery (DPA) with greater pulse pressure and mechanical deformations. In our study, maximum applied pressures lead to a reduced inversion occurrence for all wavelengths (9.7% inverted samples for the maximum pressure level compared to 13.3% and 12.1% for medium and low pressures). All levels of pressures had the least inversion at the spatial ends (+5 mm or −5 mm), except for low pressures in the positive 5 mm point. A noticeable dip in the inversion occurrence occurred with maximum strap tightness at the central position (0 mm) as seen in Figure 4f. Adjacent positions had more than twice the occurrence of inverted signals. This would agree with the theory of Kamshilin et al. [9] on the origin of PPG signals involving compression of the dermis, since the photodetector may probe the counter-phase variations adjacent to transmural pressure-induced compressions. Moreover, lateral measurements (−5, −4, −3, −2, −1 mm) during maximum pressure had a 39.6% occurrence of inversion increase in comparison to low-pressure measurements.

These results may be related to the modifications that occur in the underlying tissue during direct arterial compression. With increasing contact pressures, there will be a corresponding compression of the upper skin layers. When the PPG probe is positioned directly above the artery, this compression can even affect arterial compliance through increased tension at the arterial walls [18,19]. Since arterial compliance is improved with increasing contact pressures (until zero transmural pressure is reached [18]), there should be an increased likelihood of inversion from a wall distension perspective at maximum pressures. However, the simultaneous squeezing of the soft tissue could possibly restrict the elastic response of the connective tissue in the dermis. Maximum pressures may then create unison movements from the improved mechanical links between the arterial walls and capillary bed. This will improve the overall PPG detection, as seen in other studies of PPG signal quality and contact pressures [20,21], while also reducing inversion presence. Moreover, Nijboer and Dorlas observed the return of normal plethysmographs at locally applied pressures above 70 mmHg. Low and especially medium strap tightness still enable regions of mechanical oscillations that are in counter-phase to the arterial wall movement, but they perhaps do not assert similar restriction to dermal elasticity as during higher applied pressures. Inversion thus could occur more frequently close to the artery center for medium and lower pressures, as seen in Figure 4. Zareen et al. [22] found the mean diameter of the DPA to be around 3.32 ± 0.91 mm in the right foot of males. Assuming measurements were precisely aligned with the location of the DPA, moving 1 mm medially would still include the artery within the sensitivity volume of the measurement. Whenever the PPG probe is slightly offset to the artery center, there will be regions of the artery that do not experience similar mechanical forces. As such, a contrast in the mechanical links between the capillary bed and the artery may be established [9]. This difference might enhance the counter-phase response since one area of the artery will impede dermal elasticity more than the other. Moreover, arterial compliance will also be improved without dominating dermal structure. The occurrence of inversion might therefore increase at the neighboring positions of the artery. Veins are more compliant than arteries, and in any scenario where arteries are affected, there is likely to be a compression of the veins nearby. Venous blood would then be pushed away longitudinally toward the neighboring regions. Nijboer’s hypothesis would then also be valid. Although these observations are different from Bonnet’s findings of inversion at the strongest tonometric position above the carotid artery, it is possible that different morphological conditions, such as intensity of arterial wall movements, local tissue properties, size and compliance of blood vessels and direction of blood flow (towards the brain in arteries, toward metatarsal space in the foot), alter inversion conditions.

### 4.4. Inversion Related to Depth of Optical Path

The difference between the LEDs with respect to inversion is either caused by the location in tissue that causes inversion (inverted region), varying penetration depths, or source-detector distances on the optical platform. In Figure 4, it is clear that red/IR and green/blue pairs show an accompanying trend. In instances when red and IR are inverted, the region causing inverted samples is most likely deeper than the penetration depth of green and blue. Examples are the deep dermal or subcutaneous layers of the dorsum from where green and blue photons do not survive the ‘round trip’ back to the detector. Likewise, if blue and green wavelengths produce inverted signals but are not shown in red and IR, it is possible that the inversion region is within the upper mm layers of the dorsum. Near IR and red photons will traverse this area with sufficient remnant energy to interact with deeper layers, which will have normal optical conditions inversely matching the arterial blood pressure curve. This would be a valid explanation as to why only segments of signals can be inverted, since the presence of inverted regions would depend on depth and therefore responsiveness to the arterial expansion. The location and spatial reach of inverted regions can therefore vary, especially when considering the penetration depth of blue wavelengths of around 1 mm in tissue [23]. The origin of blue PPG signals has been speculated to stem from the non-vascularized epidermis [6]. In this case, inverted blue signals should stem from the same tissue region. Reflections from oriented erythrocytes and arterial walls cannot directly explain these blue inverted signals as there would be no photon-erythrocytes interactions. Since inverted blue signals occurred at all pressure levels, the argument of an improved propagation depth from the compressed epidermis is unlikely to be the cause. Considering the elasticity of the epidermis [24], it might lay the foundation of adjacent counter-phase areas like the capillary bed. If this is true, it also reinforces the idea of inverted green signals originating from mechanical deformations [9], due to their similarities in inversion occurrence.

An additional consideration is the source-detector distance. The IR/red and green/blue pair of LEDs are approximately positioned from the photodetector by 4.27 mm and 2.1 mm, respectively. Since photons usually take a banana-shaped path toward the detector in reflection modality [25], it is possible that regions causing inversion will be undetectable for the green and blue LED pair due to their narrow spacing. However, if the inverted region is directly below the IR or red LEDs, the argument that these photons would continue to interact with in-phase tissue would remain valid. Within the LED pairs there is also a horizontal spacing of 1.3 mm. Whether this offset had any impact on the occurrence of inversion is difficult to distinguish from Figure 4 since the LEDs are pairwise similar.

The red LED signals experienced the most inversion out of all LEDs. It is also the LED with the highest contrast in absorption coefficient in saturated and non-saturated hemoglobin (Hb). Hb has approximately an order of magnitude larger molar extinction coefficient than Hb02 in the 660 nm range [26]. Red wavelengths are therefore more sensitive to venous interference of the measurement site compared to other LEDs. This strengthens the argument that veins are involved in forming inverted signals since inversion was most present for red wavelengths. Effects, such as venous pulsations, would also impose a higher contribution to the PPG signal [27] than for IR. These pulsations are related to cardiac, respiratory, and autonomic physiological functions and could be the reason why partial inversion or altered signals were more present for red wavelengths.

### 4.5. Interindividual Variations

The frequency of inversion was highly individual. Three of the twelve participants had 29.6%, 31.1%, and 37.9% of their samples inverted. Two participants had no inverted samples. This result points toward inversion being related to individual physiology or anatomy. It was observed (although not systematically) during measurements that those with the most inversion occurrence all had a strong peripheral pulse, easily palpable at the foot. If inversion is indirectly related to the movement of the arterial walls as proposed by [9], factors such as blood pressure, elastic arteries, and good peripheral vascular health would play a role. The size of the inverted region would also be linked to the amplitude of these motions, as greater movements would affect more of the adjacent dermis. These individuals were also more likely to have inversion over all LEDs, which could be explained by the intensity of the pedal pulse. Another consideration is blood vessel layering. If the dorsalis vein is located above the DPA it might increase the occurrence of inversion from a venous engorgement standpoint. Studying the table of force measurements, none of these participants experienced any outliers with respect to contact pressure or skin surface temperature. Room temperatures were relatively stable, ranging from 21.2 to 24.6 °C (mean 22.9 °C).

Nijboer and Dorlas [7] reported that the inversion effect never occurred in the transmission modality. Although this pilot study did not study transmission PPG, a possible explanation of the inversion absence can be extrapolated from these results. Taking into consideration that inverted regions are thought to be spatially restricted, related to arterial wall movements and/or dependent on venous engorgement, the absence of inverted transmission PPG signals is caused by either (a) inverted regions tend to cause backscattering or reflection, or (b) photons that cross inverted regions continue to interact with tissue of normal optical conditions before being detected. In both scenarios, only the DC (non-pulsative level) information will be reduced. Since transmission PPG is often applied to the tip of extremities, there is an absence of large blood vessels. The likelihood of counter-phase varying areas and blood pooling is therefore lowered.

Analysis of the occurrence of inversion (Figure 4) thus suggests that PPG signal inversion during measurement above the DPA is likely to originate from mechanical deformation for blue/green wavelengths, while erythrocytes or wall reflections in combination with venous engorgement increases the likelihood for red/IR wavelengths. Table 3 presents a comparison of conclusions made in this paper, with previous papers discussing the topic.

### 4.6. Considerations of the Algorithmic Inversion Quantification

Our inversion detection algorithm can be affected by different sources of variation in the signal. The respiratory signal component, posture-related differences, and motion artifacts are the primary factors that may affect the inversion estimate. The inversion algorithm can interpret noise as systolic tails, giving uncertainty to the inversion estimate. However, close to zero inversion estimates were therefore visually verified to check whether inversion or noise was the source of the indecisive estimate. All affected samples were excluded before analysis and did not affect the results in Figure 4. Sensor-related noise, such as ambient light and channel crosstalk, is handled by signal processing integrated within the MAX86916 multi-wavelength PPG sensor and is not considered an issue for the algorithm performance. Because the inversion algorithm evaluates the AC component, it has in addition the potential to be used as an algorithm for signal quality assessment with respect to inversion artifacts. Current methods utilize pulse wave characteristics to generate indices reflecting signal quality. Once the index is computed, it remains to determine which values of this index are associated with good/poor quality PPG signals. These thresholds are acquired either by physiological thresholds or data fusion [28]. Physiological thresholds and heuristics compare extracted values to existing physiological knowledge or empirical evidence. Data fusion combines multiple signal features, such as in machine learning or probabilistic techniques, to create robust thresholds. In this inversion algorithm, a simple inversion estimate threshold would suffice. If the inversion estimates are negative, the sample is likely to contain inverted components. Changing of probe position and applying pressures could then be recommended, depending on the wavelengths utilized.

### 4.7. Study Limitations

For pragmatic reasons, only reflective mode PPG was included in this pilot study. According to Nijboer and Dorlas [7], the inverted waves are not seen in transmission PPG. This could suggest that the problem of distorted waves may be solved by using transmission instead of reflective PPG. Transmission PPG is, however, limited to thinner sites/digits, such as the fingers, and impractical for other locations, such as the dorsalis pedis or carotid arteries. Comprehension of signal anomalies during reflectance mode is therefore important if PPG is to be utilized in the assessment of pulse transit time and vascular aging in larger arteries [29], supporting the importance of studying this phenomenon for reflective PPG. The results revealed a high interindividual variation compared to the other factors influencing the occurrence of inversion, necessitating a higher sample size for precise estimates and statistical analysis of the inversion position dependency at different levels of the factors studied. In addition, the repeatability over time and reproducibility over different measurement conditions is unknown.

Another limitation of the measurement protocol was the localization of the DPA, found by palpation of the peripheral pulse, and there is a chance that the original (zero) position of the sensor could be offset by a couple of mm in either direction. Moreover, if the asymmetric position dependency seen in Figure 4 is caused by the relative position of the dorsalis vein, this result is likely influenced by variation in individual anatomical vasculature.

The optical platform introduces intrinsic spacing between the LEDs themselves and the photodetector. Despite the mm scale, these offsets do introduce spatial bias. Since one possible explanation of the inversion utilizes proximate variations in mechanical links between the capillary bed and arteries, these offsets may have affected the inversion occurrence distribution. A possible solution would be to improve the spatial resolution of the protocol with smaller medial/lateral increments and a comparison of adjacent positions could then be insightful. The schedule of sensor position changes was in a defined order, equal to all participants, of incremental steps to the medial and lateral sides of the artery. We can therefore not exclude a bias in results due to this ordering of measurements, as compared to a random schedule of positions.

A limitation of the triangulation method is the need for local troughs and peaks between pulse candidates. If the signal of interest is free of any such points, the pulses in question will not have their angles calculated. If the entire pulse sample lacks intermediate fiducial points, the triangulation algorithm is invalid, and only the symmetry estimates are evaluated.

The inversion algorithm utilized a degree of inversion threshold of 0.25 to separate inverted and non-inverted samples in this binary classification. This number was chosen from experience, and tweaking its value would affect results. The lower the threshold value, the stricter the algorithm will be, and vice versa.

## 5. Conclusions

PPG signal inversion is rarely reported in the literature, and it may not be an issue in most study cases. Our pilot study indicates that signal inversion or signal distortion can occur above the DPA artery in the foot in most healthy subjects. This can be observed at different pressures and different PPG LED wavelengths/LED to detector distances, and it appears to be sensitive to the sensor position, with respect to the artery, in a matter of only a few millimeters. The occurrence of inversion was reduced for the shorter wavelength Blue and Green LED signals, which also had narrower LED-detector separation. However, this superficial measurement may not be optimal for the examination of arterial properties where deeper light penetration depths are desired. This may have implication for peripheral vascular measurements using PPG at the dorsum of the foot either for pulse wave analysis where the shape of the signal is of interest, or for pulse arrival or transit time measurement using PPG where the sharp identification of fiducial timing points is required.

An important next step is to elucidate the conditions for inversion or signal distortion for PPG measurement at points of distal arteries. First, due to the large intersubject variability observed in our pilot study, we now appreciate a larger sample size would be needed and it could be advantageous to study both genders across a wide age range to explore vascular aging in this context. Ultrasound imaging could be used to provide a more exact determination of the DPA location for each individual, as well as the position of the dorsalis vein to account for individual variation in its position relative to the artery. The addition of measurements of ECG and systemic blood pressure will allow quantification of timing/phase shifts in the PPG signal and account for variations in blood pressure, in particular the pulse pressure acting on the tissue surrounding the artery. These improvements would benefit the understanding of the occurrence of inversion and waveform distortion potentially reducing error sources in current and future PPG applications for assessments of the peripheral vascular system.

## Figures and Tables

**Table 1:** Overview of all measurements collected for each participant, according to the position relative to the artery (+ in the medial/- in the lateral direction) and the levels of pressure from the probe on the skin.

Pressure Level	Position Medially+/Laterally Relative to Artery Central Line [mm]										
Low	−5	−4	−3	−2	−1	0	1	2	3	4	5
Medium	−5	−4	−3	−2	−1	0	1	2	3	4	5
High	−5	−4	−3	−2	−1	0	1	2	3	4	5

**Table 2:** The mean levels of force exerted on the skin from the sensor probe at the three different pressure levels and the skin/room temperature for all study participants.

Participant	Fmax [mmHg]	Fmed [mmHg]	Fmin [mmHg]	°C_Skin_	°C_Room_
S1	55.0	21.3	15.6	34.1	21.8
S2	40.9	15.3	7.7	31.1	21.2
S3	34.0	14.0	8.3	32.2	23.3
S4	51.6	29.8	21.0	32.3	22.5
S5	39.7	21.3	12.3	34.1	23.7
S6	40.6	15.7	10.2	28.9	23.5
S7	51.1	37.6	9.3	32.1	22.6
S8	35.9	16.8	3.2	36.2	24.3
S9	36.0	16.3	5.6	31.9	24.6
S10	35.7	32.6	24.5	31.1	22.5
S11	42.2	38.0	13.8	32.4	22.5
S12	47.1	27.5	17.3	35.3	22.7
Mean ± SD	42.5 ± 6.8	23.8 ± 8.5	12.4 ± 6.1	32.6 ± 1.9	22.9 ± 0.9

**Table 3:** Comparison of study findings with papers previously discussing PPG signal inversion. Findings are extracted from the study’s conclusions or sections discussing inversion.

Study Author	Findings
Weinman et al. [11]	“Inversion occurs when reflection from vessel expansions dominates the attenuated backscattered and reflected light from the surrounding tissue”.
Nijboer et Dorlas [7]	“Inversion is caused by a relative increase in optical density of the surrounding tissue. In the finger, it is brought about by an interaction between venous engorgement and application of pressure”.
Kamshilin et al. [9]	“’Pulsatile transmural pressure of the arteries, which compresses/decompresses the density of capillaries in the dermis, thus modulating the blood volume in the capillary bed’ may cause inversion due to variations on mechanical connections to the arterial walls and dermis”.
Amelard et al. [12]	“The inverted blood pulse waveform shape was consistent with the jugular venous pulse (JVP) waveform. The strong negative correlation between the arterial and venous blood pulse waveforms can be attributed to the differential pressure profiles resulting from normal cardiac cycles”.
Choi et al. [13]	“The PPG inversion we observed could be related to the pressure changes between the band and the skin as the subject turns his or her wrist. In addition, the PPG sensor could move when a person turns his or her wrist”.
Long et Wan-Young Chun [10]	“Variation in path length of light and the change in absorption of the skin layers are the reasons causing inversion. The reflective factor of erythrocytes plays an essential role in the generation of the inversion effect”.
Bonnet et al. [8]	“Inverted PPG signal occurs when PPG sensors are placed at the exact same location than tonometry does (where the pulse is best felt)”.
Our work	PPG signal inversion during measurement above the DPA is likely to originate from mechanical deformation for blue/green wavelengths while erythrocytes or wall reflections in combination with venous engorgement increases the likelihood for red/IR wavelengths. Individual physiology and local anatomy seem to be a challenge for reproducibility in obtaining undistorted measurements of the DPA PPG. The sensor’s relative position to the DPA, contact pressure, and LED utilized all impact the occurrence of inversion in various degrees.

## Data Availability

The original data presented in the study are openly available on GitHub at https://github.com/FredrikWJerve/PPG-Data, accessed on 7 October 2024.
